# Thermomechanical Behaviour of Chemically Cured Polymer Composites: Preliminary Analysis of the Scale Effect

**DOI:** 10.3390/ma19102093

**Published:** 2026-05-16

**Authors:** Łukasz Suchecki, Szymon Arkanowicz, Krzysztof Piernik, Angelika Milena Jasińska, Piotr Zagulski

**Affiliations:** 1 Faculty of Electrical Engineering, Czestochowa University of Technology, Al. Armii Krajowej 17, 42-200 Czestochowa, Poland; szymon.arkanowicz@pcz.pl; 2 Faculty of Mechatronics and Mechanical Engineering, Kielce University of Technology, Aleja Tysiąclecia Państwa Polskiego 7, 25-314 Kielce, Poland; piernikkrzysztof@gmail.com (K.P.); pzagulski@tu.kielce.pl (P.Z.); 3 Faculty of Management and Computer Modeling, Kielce University of Technology, Aleja Tysiąclecia Państwa Polskiego 7, 25-314 Kielce, Poland

**Keywords:** composites, scale effect, L-RTM, tensile testing, DSC

## Abstract

This study examines the influence of scale effects on the thermomechanical and structural performance of chemically cured, glass-fibre-reinforced polyester composites. Two reinforcement architectures—plain 0/90° fabric and biaxial fabric—were analysed to assess differences in resin flow, curing behaviour, and mechanical characteristics. Differential Scanning Calorimetry (DSC) was employed to characterise cross-linking kinetics at 15 °C, 19 °C, and 25 °C, demonstrating that higher cure temperatures markedly accelerate gelation and cross-linking. Composite plates were manufactured by Light Resin Transfer Moulding (L-RTM), and static tensile tests were conducted in accordance with PN-EN ISO 527-4. The results confirm that reinforcement architecture strongly affects processability and mechanical performance. The 0/90° fabric provided superior resin permeability and shorter infusion times, whereas the biaxial fabric required higher injection pressure and exhibited longer curing duration. Statistical analysis based on Weibull’s brittle strength theory verified the presence of scale effects: larger specimens displayed lower nominal strength due to a higher probability of internal flaws. Multiple regression modelling further revealed relationships between geometric and mechanical parameters: maximum (destructive) stress, Rm, was the dominant factor influencing both specimen thickness and number of layers, while deformation at maximum stress (εm) primarily determined specimen length. These findings highlight the necessity of accounting for size-dependent behaviour when designing and testing polymer composites. Considering scale effects enables more reliable extrapolation from laboratory-scale tests to full-scale components, thereby improving predictability and structural reliability in engineering applications.

## 1. Introduction

The performance of chemically cured polymer composite systems is governed by the interplay between curing kinetics, the evolving microstructure, and subsequent thermomechanical loading conditions. During cure, thermosetting matrices undergo irreversible cross-linking reactions accompanied by volumetric shrinkage, development of internal stress, and changes in thermal and mechanical properties [1,2,3]. Consequently, even for nominally identical chemistries and cure cycles, the final state of the cured part may exhibit significant heterogeneity in properties and residual stress fields.

Under thermomechanical loading—elevated temperature, cyclic stress, or combined thermo-mechanical strain—the service behaviour is influenced by matrix properties (e.g., modulus, glass transition temperature, and yield/fracture characteristics), as well as by residual cure-induced stresses and microstructural defects such as voids and micro-cracks [4,5]. A critical consideration in materials engineering is the impact of scale—in terms of specimen size, reinforcement dimensions, or structural geometry—on measured or effective properties. The scale effect posits that nominal strength, stiffness, or fracture energy may not scale linearly with structural dimension. Larger volumes or features often exhibit reduced nominal strength and altered failure modes due to statistical and energetic factors [2,6,7,8,9].

The Weibull size effect describes the relationship between specimen dimensions and strength, resulting from the statistical distribution of defects within the material. In composite laminates—where failure is initiated by microvoids, local fibre misalignments, and matrix inhomogeneities—increasing the loaded volume raises the probability of encountering a critical flaw, leading to a reduction in measured strength [10].

Studies on fibrous composites confirm that mechanical properties strongly depend on specimen length, and the scatter of results increases with sample size [11]. Wisnom showed that the size effect is particularly pronounced in textile laminates, where complex microstructural arrangements produce higher variability in strength [12,13]. Similar trends have been observed in unidirectional laminates, where longer specimens exhibit reduced nominal strength [14].

Therefore, incorporating the Weibull size effect is essential for analysing laminate strength and extrapolating laboratory data to larger structural components as it influences the interpretation of experimental results and the modelling of damage mechanisms [15].

In chemically cured polymer composites, scale effects may manifest through

(1) Smaller specimens exhibiting higher strength/stiffness due to a lower probability of critical flaws;
(2) Variation in cure shrinkage-induced residual stress with part thickness, altering stress magnitudes and gradients;
(3) Size-dependent microstructure (e.g., voids or micro-damage) modifying fatigue life, creep response, or stiffness degradation compared with inferences from small-scale tests.

Microscale studies on thermosets have shown strength up to an order of magnitude higher than corresponding macroscale measurements, alongside reduced fracture energy, evidencing size-dependent behaviour in cross-linked networks [10]. Despite its clear importance, a substantial proportion of polymer composite studies still consider a single specimen size with limited systematic variation under combined thermal and mechanical loadings [6,7]. This gap is particularly consequential for high-performance applications (e.g., aerospace and automotive), where component dimensions span orders of magnitude and experience fluctuating temperatures post-cure [8]. A systematic assessment of thermomechanical behaviour across sizes and geometries is therefore warranted.

Accordingly, this study analyses the thermomechanical response of chemically cured polymer composites of different sizes to elucidate how scale influences post-cure mechanical and thermal performance. Specifically, it investigates interactions among cure-induced residual stress, dimensional effects, and subsequent thermomechanical loading and their impact on stiffness, strength, and deformation behaviour. The overarching objective is to support more reliable extrapolation from laboratory data to full-scale components, enhancing predictability in real-world applications.

## 2. Materials and Methods

This chapter discusses the materials used to conduct the experiment and their parameters. The subsections also describe the methods used to produce the samples and the parameters of the methods.

### 2.1. Materials

**Figure 1:**
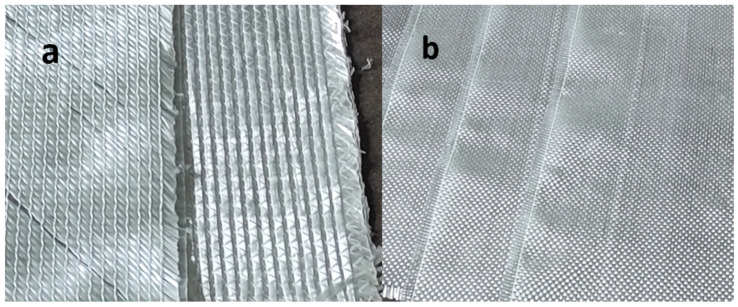
(**a**) Biaxial glass fabric mat; (**b**) 0/90 glass mat.

Composite panels were manufactured from an unsaturated polyester resin using two types of glass fibre reinforcements differing in areal weight and number of layers. Prior to manufacturing, DSC tests were performed on the polyester resin to identify cross-linking parameters; the results were compared with the manufacturer’s data (Havel Svésedlice 67, 783 54 Přáslavice, Czech Republic), summarised in Table 1. Composites were produced by the Light Resin Transfer Moulding (L-RTM) method. Two reinforcements were used: biaxial glass fabric with an areal weight of 450 g·m^−2^ (Figure 1a) and 0/90° glass fabric with an areal weight of 160 g·m^−2^ (Figure 1b). Relevant reinforcement parameters are given in Table 2. The matrix was Havelpol 1 polyester resin, cured with 2% Butanox M50 (by resin mass).

Beyond fibre arrangement, the reinforcements differ in areal weight. Under principal stresses parallel to the loading direction, the biaxial architecture demands a higher areal weight than the 0/90° fabric to achieve comparable load-bearing capability. Owing to reinforcement orientation, the materials differ in Young’s modulus and elongation at break. Reinforcement architecture also significantly affects resin flow during manufacturing. For good flow, the 0/90° orientation is advantageous, offering less resistance to resin permeation from four sides. Biaxial fabric exhibits lower permeability, requires higher injection pressure, and has a significantly longer permeation time than 0/90° fabric [9].

### 2.2. Methods

In this subsection, we will discuss the parameters of Differential Scanning Calorimetry tests, static tensile tests, and sample production using the L-RTM method.

#### 2.2.1. Differential Scanning Calorimetry Method

DSC testing. DSC was performed using a Netzsch DSC 204 Phox (Gebrüder-Netzsch-Straße 19, 95100 Selb, Germany) heat-flux instrument at a temperature range of −200 °C to 600 °C. A 12 mg resin sample was sealed in an aluminium crucible. One identification scan was run from 0 °C to 220 °C, and three isothermal cross-linking tests were conducted at 15 °C, 19 °C, and 25 °C, each for 180 min [16,17,18,19].

#### 2.2.2. Production of Samples for Testing

**Figure 2:**
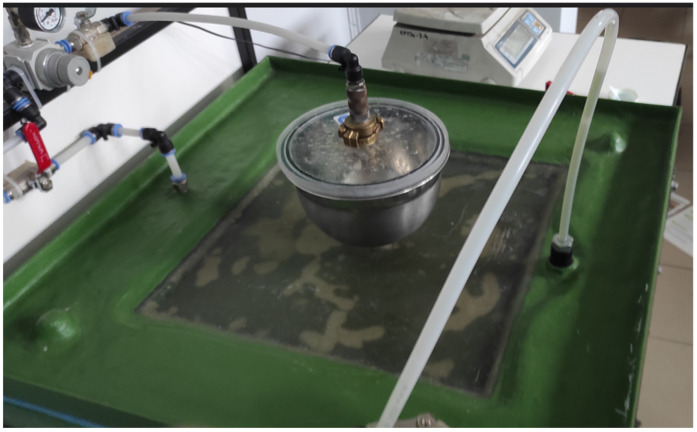
L-RTM mounding.

After DSC, composite panels were produced using the L-RTM vacuum method (Figure 2), which provides high repeatability and a low defect rate. To evaluate the relationship between defect population and strength, additional panels were produced for comparison.

#### 2.2.3. Static Tensile Test

**Figure 3:**
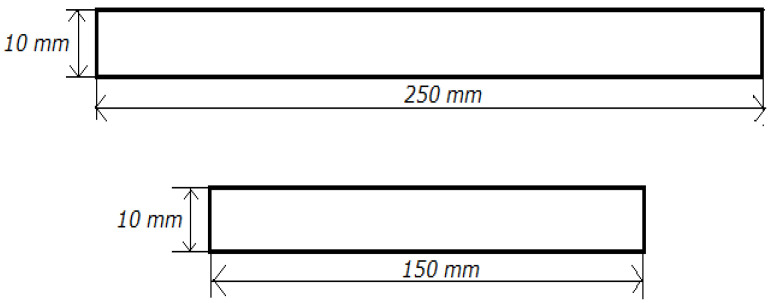
Dimensions of samples accepted for static tensile testing, taking into account the scale effect.

Panels were cut to the dimensions shown in Figure 3. Static tensile tests were performed according to PN-EN ISO 527-4 [20] using a Galdabini Quasar 25 (183 Via Giovanni Xxiii, Cardano Al Campo, Lombardia 21010, Italy) test machine equipped with an extensometer. Standardised specimens (Figure 3). he test specimens had dimensions of 10 mm × 150 mm and 10 mm × 250 mm. They were tested under displacement control at 50 mm·min^−1^, and at 1 mm·min^−1^ when an extensometer was used [21]. Based on the data dispersion (standard deviation), the influence of thickness on strength and the evolution of defects with scale were assessed [20,22].

In order to ensure consistent nomenclature in the analysis, the material sets were assigned the following codes. Furthermore, the specimens were grouped according to material configuration, length, and number of layers, thereby enabling a clearer presentation and comparison of the results.

Types are biaxial (Bi) or 0/90 (090), length (250 or 150), and layers (4 or 2):

1-Biaxial, 150 mm, 2 layers (Bi.150.2); 4-Biaxial, 150 mm, 4 layers (Bi.150.4)

2-Biaxial, 250 mm, 2 layers (Bi.250.2); 5-0/90, 250 mm, 4 layers (090.250.4)

3-Biaxial, 250 mm, 4 layers (Bi.250.4); 6-0/90, 150 mm, 4 layers (090.150.4)

## 3. Results

This section presents (i) DSC characterisation of gelation and cross-linking enthalpy; (ii) tensile properties—including maximum stress, longitudinal modulus, and deformation at maximum stress; and (iii) statistical analysis of scale effects.

### 3.1. Thermal Testing Using Differential Scanning Calorimetry

**Figure 4:**
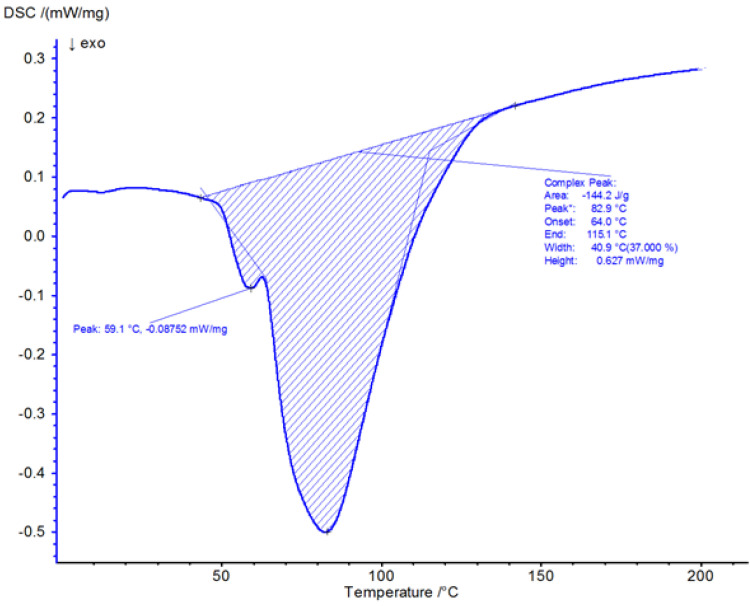
DSC thermogram of resin from 0–220 °C.

**Figure 5:**
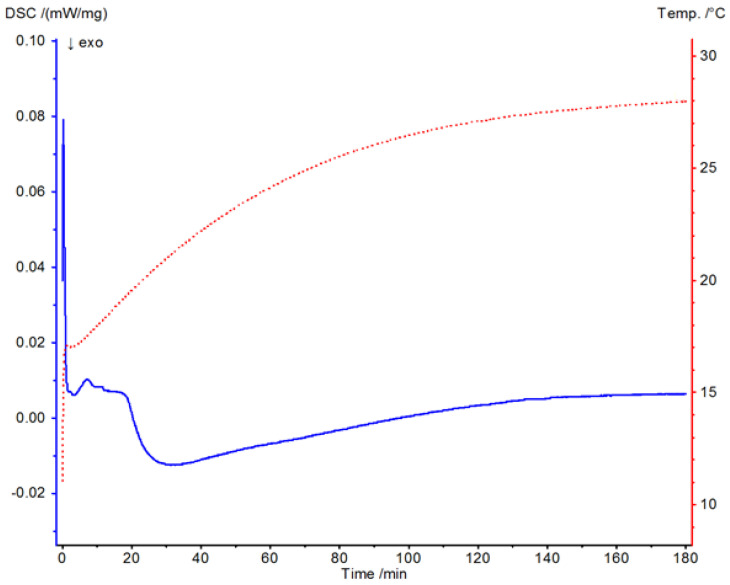
DSC thermogram of resin from a temperature of 15 °C.

**Figure 6:**
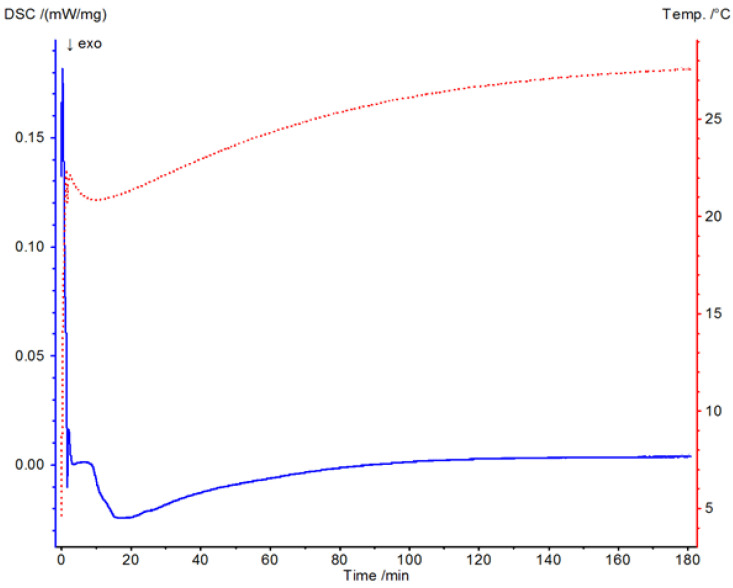
DSC thermogram of resin from a temperature of 19 °C.

**Figure 7:**
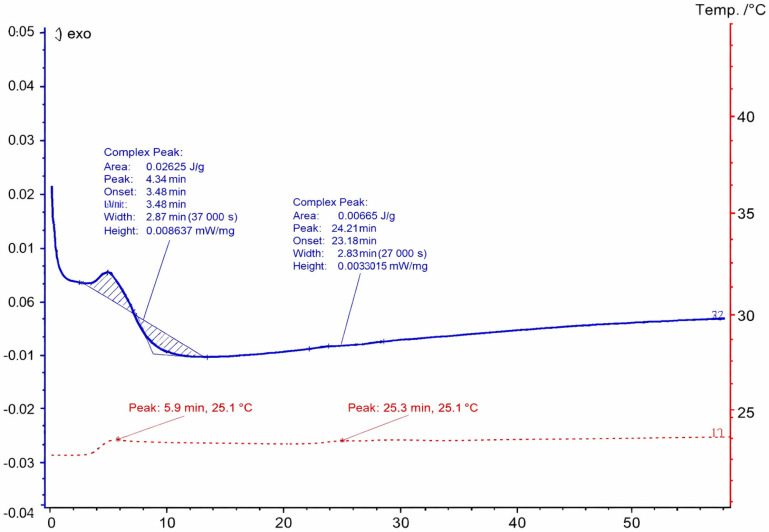
DSC thermogram of resin from a temperature of 25 °C.

Figure 4 presents the DSC thermogram of Havelpol1 polyester resin recorded over the temperature range of 0–220 °C. Figure 5, Figure 6 and Figure 7 illustrate the isothermal behaviour of the resin at 15 °C, 19 °C, and 25 °C, respectively. An exothermic peak was recorded, with enthalpy of 144.2 J·g^−1^. The reaction onset was 64.0 °C and completion was 115.1 °C; the peak temperature was 82.9 °C. These values were compared with a reference polyester resin from the Netzsch database (Proteus ver. 8.2).

At 15 °C, the heat flow baseline remained at ~0 mW·mg^−1^ between 140 and 180 min, decreased to −0.01 mW·mg^−1^ from 140 to 30 min, and exhibited two increases (to ~0.01 and 0.08 mW·mg^−1^) between 30 and 0 min. Transient disturbances at 0–0.2 min reflect sample insertion. The sample transitioned from liquid to gel over ~0.2–20 min and commenced cross-linking at ~20–30 min, with temperature peaks observed from ~40–150 min. The resin was fully cross-linked only in the final interval, ~150–180 min.

At 19 °C, heat flow was ~0 mW·mg^−1^ between 100 and 180 min, decreasing to −5 × 10^−3^ mW·mg^−1^ from 100 to 20 min. Two increases (to ~0 and ~0.2 mW·mg^−1^) were observed from 20 to 0 min. After insertion transients (0–0.2 min), the sample progressed from liquid to gel over ~0.2–10 min and began cross-linking. Temperature peaks appeared at ~10–20 min, and subsequent cross-linking stages persisted from ~20–120 min. Full cross-linking occurred during the period of ~120–180 min.

At 25 °C, with a shorter observation window, heat flow was −5 × 10^−3^ mW·mg^−1^ over ~40–50 min, decreasing to −0.01 mW·mg^−1^ from ~40 to 10 min, and then rising was performed twice to ~5 × 10^−3^ and ~0.02 mW·mg^−1^ from 10 to 0 min. After insertion transients (0–0.2 min), the sample gelled at ~0.2–0.4 min and initiated cross-linking at ~0.4–0.6 min. Temperature peaks appeared at ~0.6–10 min, with subsequent cross-linking stages observed at up to ~5.8–10 min. Full cross-linking occurred by ~5.8–60 min.

Summary: Increased curing temperature substantially accelerated gelation and completion of cross-linking.

### 3.2. Mechanical Properties

Composite material samples with varied layer structure and geometry, made of two types of reinforcing fabrics: classic fabric with a 0/90° fibre arrangement and biaxial fabric, were analysed. The use of different fibre orientations made it possible to examine the effect of structural anisotropy on mechanical properties. Biaxial fabrics exhibit increased ability to transfer multidirectional loads, while the 0/90° arrangement is characterised by a more predictable material response along the main load axes. The samples also differed in the number of layers (2 or 4) and length (150 mm and 250 mm), which allowed the assessment of the impact of laminate thickness and sample slenderness on its mechanical response.

#### 3.2.1. Maximum Stress

**Figure 8:**
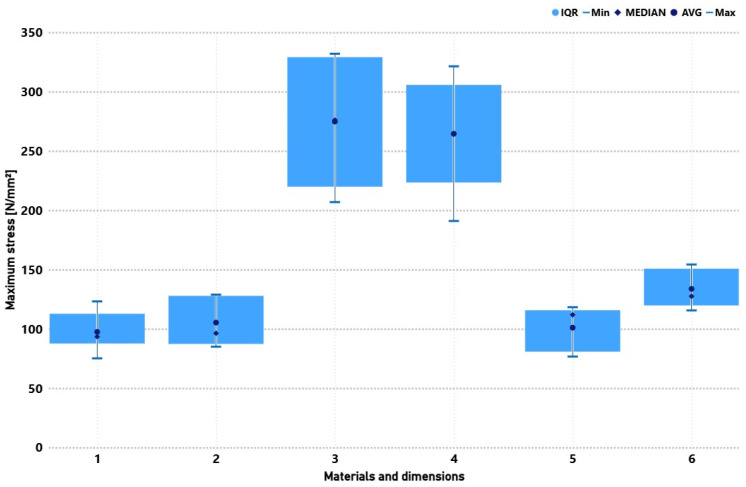
Comparison of mechanical properties (maximum stress) for six groups of composite specimens with varying reinforcement type, specimen length, and number of layers. Specimens: 1 (Bi.150.2), 2 (Bi.250.2), 3 (Bi.250.4), 4 (Bi.150.4), 5 (090.250.4), and 6 (090.150.4).

Figure 8 presents the maximum strains recorded for the tested composites. The highest load-bearing capacity was achieved by biaxial, 4-layer specimens, regardless of length, with average stresses exceeding 260 N·mm^−2^ and maxima around 300 N·mm^−2^. Reducing the layer count to two lowered strength to ~100–120 N·mm^−2^, confirming the dominant role of fibre volume fraction in load transfer. The 0/90° fabric, while markedly weaker (130–140 N·mm^−2^ for 4 layers), exhibited lower result variability, suggesting higher repeatability and structural homogeneity. The effect of specimen length was secondary; increasing from 150 mm to 250 mm slightly increased scatter.

#### 3.2.2. Longitudinal Elasticity Module

**Figure 9:**
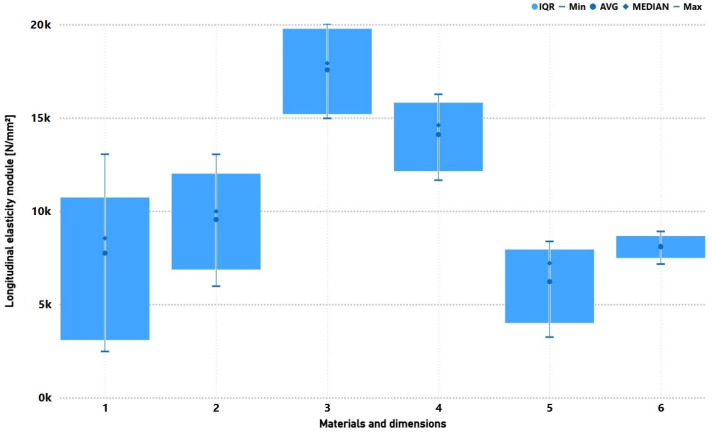
Comparison of mechanical properties (longitudinal elasticity module) for six groups of composite specimens with varying reinforcement type, specimen length and number of layers. Specimen groups: 1 (Bi.150.2), 2 (Bi.250.2), 3 (Bi.250.4), 4 (Bi.150.4), 5 (090.250.4), and 6 (090.150.4).

Figure 9 presents the longitudinal elasticity of the tested composites. The highest moduli were obtained for four-layer biaxial specimens (~17–18 × 10^3^ N·mm^−2^). For 0/90°, the values were nearly half (~8–9 × 10^3^ N·mm^−2^). Fewer layers reduced stiffness to ~6–10 × 10^3^ N·mm^−2^. Longer specimens exhibited greater modulus variability, likely due to deformation localisation and earlier damage initiation over longer gauge lengths.

#### 3.2.3. Deformation at Maximum Stress

**Figure 10:**
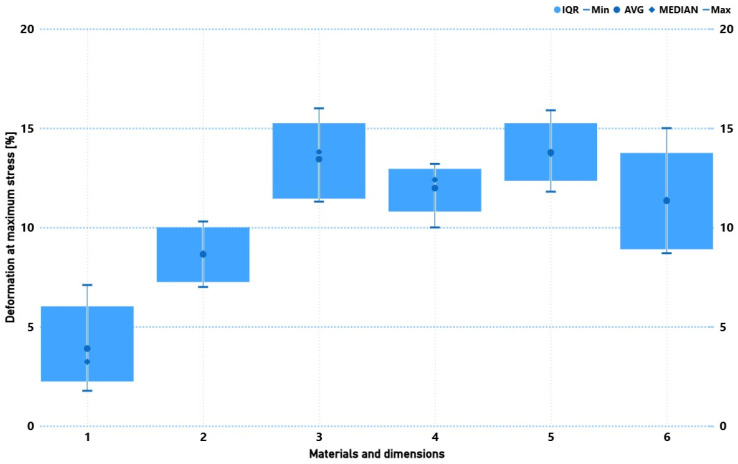
Comparison of mechanical properties (deformation at maximum stress) for six groups of composite specimens with varying reinforcement type, specimen length, and number of layers. Specimen groups: 1 (Bi.150.2), 2 (Bi.250.2), 3 (Bi.250.4), 4 (Bi.150.4), 5 (090.250.4), and 6 (090.150.4).

Figure 10 presents the deformation at maximum stress for the tested composites. Two-layer, 150 mm specimens showed the lowest deformation (mean 3.9%, max ~7.1%), indicating rapid approach to a critical state with limited deformation capacity. Increasing length to 250 mm raised average deformation to ~8.6%. Increasing to four layers substantially improved deformability (means ~12–14%, maxima up to ~16%), evidencing greater elastic energy accumulation before failure in thicker laminates.

Comparing architectures, four-layer biaxial and 0/90° systems had similar mean deformation, but 0/90° laminates displayed greater spread between minima and maxima, potentially reflecting micro-cracking and delamination transverse to fibres. High upper-quartile values (Q75 up to ~15.25% for biaxial and ~13.75–15.25% for 0/90°) indicate quasi-plastic behaviour near failure.

### 3.3. Statistical Analysis

To quantify scale effects, multiple regression was used to evaluate how geometric parameters influence strength indicators. The hypothesis is that resistance to crack initiation and propagation depends not only on microstructure but also on loaded element size. Table 3, Table 4, Table 5, Table 6, Table 7 and Table 8 present the results of the statistical analysis and the regression models.

#### 3.3.1. Regression Model with Sample Thickness

The model (predictors: Et, εm, Rm) yielded R^2^ ≈ 0.461; F = 11.704; *p* < 0.00001. Only Rm was statistically significant (b ≈ 0.01237; β* ≈ 0.797; *p* ≈ 0.00098), indicating a strong positive association between maximum stress and thickness. Deformation at maximum stress (β* ≈ −0.228; *p* ≈ 0.102) and modulus (β* ≈ −0.032; *p* ≈ 0.875) were not significant.

Thickness = 2.7568 − 0.00001∙Et − 0.06453⋅ε_max + 0.01237⋅Rm(1)

**Table 3:** Basic statistics for the dependent variable thickness.

Statistics for Thickness	Value
R multiple	0.67921
Multiple R^2^	0.46132
Adjusted R^2^	0.42190
F (3.41)	11.70398
*p*	0.00001
Standard estimation error	0.89946

**Table 4:** Regression of the independent variable (thickness predictor).

Regression Model for Thickness (N = 45)	b*	Error. Std. (z b*)	b	Error. Std. (z b)	t(41)	*p*
W. free			2.75680	0.42824	6.437513	0.00000
Longitudinal elasticity module [Et] [N/mm^2^]	−0.03235	0.20449	−0.00001	0.00006	−0.158221	0.87506
Deformation at maximum stress [εm] [%]	−0.22761	0.13623	−0.06453	0.03862	−1.670802	0.10238
Maximum stress [Rm σm] [N/mm^2^]	0.79710	0.22449	0.01237	0.00348	3.550680	0.00098

#### 3.3.2. Regression Model with Sample Length

The model gave R^2^ ≈ 0.434; F ≈ 10.472; *p* ≈ 3.04 × 10^−5^. The strongest predictor was εm (b ≈ 9.248; β* ≈ 0.768; *p* < 0.00001), confirming a robust positive correlation: specimens with greater elongation were significantly longer. Rm was also significant with a negative effect (b ≈ −0.4049; β* ≈ −0.614; *p* ≈ 0.0108). Et did not reach significance (b ≈ 0.004218; β* ≈ 0.358; *p* ≈ 0.0949).

Length = 126.667 + 0.004218∙Et + 9.2482⋅ε_max + 0.4049⋅Rm(2)

**Table 5:** Basic statistics for the dependent variable length.

Statistics for Length	Value
R multiple	0.65865
Multiple R^2^	0.43383
Adjusted R^2^	0.39240
F (3,41)	10.47195
*p*	0.00003
Standard estimation error	39.17076

**Table 6:** Regression of the independent variable (length predictor).

Regression Model for Length (N = 45)	b*	Error. Std. (z b*)	b	Error. Std. (z b)	t(41)	*p*
W. free			126.667726	18.6495308	6.79200602	0.00000
Longitudinal elasticity module [Et] [N/mm^2^]	0.35841	0.20964	0.004218	0.00246713	1.70962208	0.09490
Deformation at maximum stress [εm] [%]	0.76793	0.13966	9.248235	1.68194457	5.49853731	0.00000
Maximum stress [Rm σm] [N/mm^2^]	−0.61439	0.23015	−0.404911	0.15167937	−2.6695166	0.01084

#### 3.3.3. Regression Model with Sample Number of Layers

The model explained ~27% of variance (R^2^ ≈ 0.272; F ≈ 5.116; *p* ≈ 0.00424). Only Rm was significant (b ≈ 0.01544; β* ≈ 0.529; *p* ≈ 0.049), indicating that higher destructive stress is associated with more layers (and thus increased thickness). εm (*p* ≈ 0.466) and Et (*p* ≈ 0.674) were not significant.

Number of layers = 1.7246 − 0.00005∙Et + 0.06218⋅ε_max + 0.01544⋅Rm(3)

**Table 7:** Basic statistics of the dependent variable layer.

Statistics for the Number of Layers	Value
R multiple	0.52191
Multiple R^2^	0.27239
Adjusted R^2^	0.21915
F (3,41)	5.11633
*p*	0.00424
Standard estimation error	1.96591

**Table 8:** Regression of the independent variable (layer predictor).

Regression Model for the Number of Layers (N = 45)	b*	Error. Std. (z b*)	b	Error. Std. (z b)	t(41)	*p*
W. free			1.61420	0.93599	1.72460	0.09213
Longitudinal elasticity module [Et] [N/mm^2^]	−0.10072	0.23766	−0.00005	0.00012	−0.42379	0.67393
Deformation at maximum stress [εm] [%]	0.11663	0.15832	0.06218	0.08441	0.73663	0.46554
Maximum stress [Rm σm] [N/mm^2^]	0.52929	0.26091	0.01544	0.00761	2.02864	0.04902

#### 3.3.4. Comparison of Predictor Influences

Comparative analysis indicates that Rm plays a key role in explaining thickness and number of layers (β* ≈ 0.80 and 0.53, respectively). In contrast, εm dominates the length model (β* ≈ 0.77), showing that the material’s capacity to elongate under high load conditions is the primary determinant of specimen length. Across models, Et did not achieve statistical significance, suggesting a lesser role within the tested ranges and configurations.

## 4. Discussion

The results corroborate the relevance of scale effects in chemically cured glass fibre composites, consistent with Weibull’s brittle strength distribution. The observed reduction in average strength with increasing specimen volume or layer count reflects the higher probability of critical defect presence in larger structures. These findings align with reports by Salviato et al. [2] and Liu and Luo [6], who identified size-dependent reductions in mechanical strength in fibre-reinforced and cross-linked polymer systems. Notably, the present work demonstrates that scale effects remain detectable under standard L-RTM processing using polyester matrices.

Dimensional tolerances prescribed by PN-EN ISO 527-4 strictly limit allowable deviations in specimen geometry. The regression analysis shows that even small changes in thickness or length can produce measurable differences in tensile parameters. This implies that dimensional variability, even within standard limits, may significantly affect apparent mechanical performance—underscoring the need for tight geometric control during material qualification and industrial testing.

From an application standpoint, extrapolation from laboratory-scale specimens to full-scale components must explicitly consider size effects. As tested volume increases, apparent strength decreases; naive up-scaling from small specimens can overestimate performance. For large composite structures in aerospace and automotive sectors, scale effects should be explicitly included in design allowables, strength models, and safety factors. The regression models linking Rm, thickness, and εm offer practical tools to anticipate and mitigate scale-related performance losses.

Finally, fibre architecture critically influences both processability and structural response. The 0/90° fabric affords superior permeability and shorter infusion times, whereas the biaxial fabric—despite directionally higher strength—requires higher pressure and longer cure, consistent with lower permeability. These observations emphasise that scale effects are not purely statistical; they are also technologically mediated through the coupling of geometry, fibre architecture, and process kinetics.

## 5. Conclusions

1. Cure kinetics: DSC demonstrated that increasing temperature from 15 °C to 25 °C substantially accelerates gelation and cross-linking completion in the polyester system.
2. Processability vs. Architecture: The 0/90° fabric exhibited higher permeability and shorter infusion time, while the biaxial fabric required higher injection pressure and showed longer curing duration.
3. Mechanical behaviour: Biaxial, four-layer laminates achieved the highest strengths and moduli; reducing layer count decreased both. The 0/90° laminates showed lower variability, indicating higher structural homogeneity.
4. Scale effect: Larger/thicker specimens exhibited reduced nominal strength, consistent with Weibull statistics. Multiple regression identified Rm as the dominant predictor of thickness and number of layers, while εm primarily determined specimen length. Et was not statistically significant within the tested ranges.
5. Design implications: Accounting for size-dependent behaviour is essential when translating laboratory data to full-scale structures. The models presented can inform design allowables and quality control for L-RTM polyester laminates.

## Figures and Tables

**Table 1:** Parameters of the Havelpol1 polyester resin produced by Havel composites.

	Havelpol1
Density at 25 °C	1200 kg/m^3^
Amount of catalyst	1–2%
Operating time in minutes at 15 °C	38 min
Operating time in minutes at 20 °C	26 min
Operating time in minutes at 25 °C	17 min
Viscosity at 25 °C	thixotropic

**Table 2:** Fibreglass mats manufactured by Rymatex were used to prepare composites.

	0/90	Biaxial
density	160 g/m^2^	450 g/m^2^
fibre weave	0°/90°	0°/90° +/− 45°

## Data Availability

The original contributions presented in this study are included in the article. Further inquiries can be directed to the corresponding authors.
